# Comparison of Medicare Advantage vs Traditional Medicare for Health Care Access, Affordability, and Use of Preventive Services Among Adults With Low Income

**DOI:** 10.1001/jamanetworkopen.2022.15227

**Published:** 2022-06-07

**Authors:** Rahul Aggarwal, Suhas Gondi, Rishi K. Wadhera

**Affiliations:** 1 Richard A. and Susan F. Smith Center for Outcomes Research, Beth Israel Deaconess Medical Center, Boston, Massachusetts; 2 Harvard Medical School, Boston, Massachusetts

## Abstract

**Importance:**

The Medicare Advantage (MA) program has doubled in size during the past decade, and enrollment among adults with low income has increased rapidly. Such adults face significant barriers in accessing care, leading to poorer health outcomes. Therefore, understanding how health care access, preventive care, and care affordability compare for adults with low income who are enrolled in MA vs traditional Medicare (TM) is critically important.

**Objective:**

To compare measures of health care access, preventive care use, and affordability of care between adults with low income who are enrolled in MA vs TM.

**Design, Setting, and Participants:**

This nationally representative cross-sectional study used the 2019 National Health Interview Survey to compare 2622 adults aged 65 years or older with low income who were enrolled in MA vs TM. Data were analyzed from December 5, 2021, to April 10, 2022.

**Main Outcomes and Measures:**

Measures of health care access, preventive care use, and health care affordability.

**Results:**

The study cohort included 2622 adults aged 65 years or older with low income, resulting in a weighted cohort of 14 222 243 adults, of whom 5 641 049 (39.7%) were enrolled in MA and 8 581 194 (60.3%) in TM. The overall age of the cohort was 74.6 years (95% CI, 74.3-74.9). Between the MA and TM groups, the mean age (74.5 years [95% CI, 74.1-75.0] vs 74.7 years [95% CI, 74.3-75.1]; *P* = .63) and sex distribution (63.6% women [95% CI, 59.8%-67.3%] vs 60.4% women [95% CI, 57.4%-63.3%]; *P* = .17) were similar, but adults with low income in MA were more likely to be non-Hispanic Asian (7.6% [95% CI, 5.0%-10.1%] vs 3.8% [95% CI, 2.4%-5.3%]; *P* = .01) or Hispanic (18.1% [95% CI, 14.3%-21.9%] vs 9.4% [95% CI, 7.2%-11.7%]; *P* < .001). Adults with low income in MA compared with those enrolled in TM were more likely to have a usual place of care (97.7% vs 94.9%; adjusted odds ratio [aOR], 2.37 [95% CI, 1.38-4.07]), but similarly likely to have a recent physician visit (95.5% vs 93.5%; aOR, 1.39 [95% CI, 0.88-2.17]) and to delay medical care (5.3% vs 5.7%; aOR, 0.83 [95% CI, 0.56-1.24]) or not seek medical care (5.6% vs 5.9%; aOR, 0.86 [95% CI, 0.56-1.30]) due to costs. For preventive care measures, adults with low income in MA were more likely than those in TM to have undergone a recent cholesterol screening (98.7% vs 96.6%; aOR, 2.58 [95% CI, 1.27-5.22]). However, there were no significant differences between the MA and TM groups in the likelihood of diabetes screening (90.6% vs 87.6%; aOR, 1.21 [95% CI, 0.87-1.66]), blood pressure screening (96.8% vs 95.2%; aOR, 1.37 [95% CI, 0.84-2.23]), or receipt of an influenza vaccination in the past year (66.3% vs 63.8%; aOR, 1.16 [95% CI, 0.93-1.45]). Adults with low income in MA or TM were similarly likely to be concerned about paying medical bills (47.3% vs 44.2%; aOR, 1.09 [95% CI, 0.88-1.35]) or have problems paying medical bills (17.1% vs 17.2%; aOR, 0.94 [95% CI, 0.69-1.27]) and were also similarly likely to delay filling prescriptions (7.4% vs 6.2%; aOR, 1.22 [95% CI, 0.78-1.92]) or to not fill prescriptions (7.8% vs 7.4%; aOR, 1.01 [95% CI, 0.70-1.45]) due to costs.

**Conclusions and Relevance:**

In this study of Medicare beneficiaries with low income, key measures of health care access, preventive care use, and health care affordability generally did not differ between those enrolled in MA vs TM.

## Key Points

**Question:**

How do health care access, preventive care use, and affordability of care compare between adults aged 65 years or older with low income enrolled in Medicare Advantage vs traditional Medicare?

**Findings:**

In this cross-sectional study of 2622 adults with low income, no associations for most measures of health care access and preventive care use were observed between Medicare Advantage vs traditional Medicare. These adults were similarly likely to experience financial strain from medical bills and cost-associated barriers to prescription drugs.

**Meaning:**

Although Medicare Advantage provides more generous benefits and incentives to coordinate care and manage health, there were no meaningful differences across most measures for adults with low income enrolled in Medicare Advantage vs traditional Medicare, which may have implications regarding advancing health equity in Medicare.

## Teaser

This cross-sectional study compares health care access, preventive care use, and affordability of care for adults aged 65 years or older with low income who are enrolled in Medicare Advantage vs traditional Medicare.

## Introduction

In the United States, older adults with low income who are enrolled in the Medicare program experience markedly worse health outcomes than their counterparts with higher income.^1^ These differences are due in part to the substantial barriers adults with low income face in accessing and affording health care, including preventive care and prescription drugs.^2,3^ Policy makers and health care leaders have made addressing such barriers a national priority to reduce health inequities in Medicare.^4^

Health insurance plan design is 1 potential way to improve health care access and affordability for adults with low income.^5^ Although most adults aged 65 years or older receive insurance coverage through traditional Medicare (TM), enrollment in Medicare Advantage (MA) has increased rapidly during the last decade, and approximately 42% of all Medicare beneficiaries (>26 million) now receive coverage through this program.^6^ Medicare Advantage plans receive risk-adjusted, capitated payments from the Centers for Medicare & Medicaid Services and are accountable for the costs of health care for assigned beneficiaries. Because MA plans receive fixed per-person payments for their beneficiaries, they have an incentive to control health care spending. Although this incentive could encourage stinting or rationing of necessary care, it could also lead to greater emphasis on preventive care measures and eliminate the need for more expensive forms of medical care.^7^ Prior work demonstrated that, for most Medicare beneficiaries, MA was associated with more preventive care visits, fewer hospital admissions, lower spending, and better quality of care relative to TM.^8,9,10,11^

However, to our knowledge, little is known about whether differences in health care access and affordability exist specifically among adults with low income, a vulnerable subset of the Medicare population that has a higher burden of comorbid conditions, has greater health care needs, and experiences poor health outcomes.^1,12^ Several aspects of MA may disproportionately benefit adults with low income, including the prevalence of zero-dollar premium plans, increased care coordination, and the provision of supplemental benefits, including dental or vision, transportation, and other services.^13^ Conversely, some aspects of MA plans, such as more restricted networks, may impede access to care for adults with low income and may potentially widen inequities.^13^

Therefore, in this study, we had 3 aims. First, we assessed whether access to health care differs for adults with low income enrolled in MA vs TM. Second, we evaluated whether there are differences in the use of preventive care services between these groups. Third, we compared affordability of care and prescription drug use between adults with low income who were enrolled in MA and those who were enrolled in TM. Understanding differences in health care access, preventive care, and affordability of care among adults with low income in MA vs TM is critically important as policy makers evaluate ways to improve health equity, especially since enrollment of beneficiaries with low income in the MA plans is increasing rapidly.^14^

## Methods

### Study Population

This cross-sectional study included adults with low income who responded to the 2019 National Health Interview Survey (NHIS). The NHIS is a nationally representative annual health surveillance survey conducted by the National Center for Health Statistics. The survey includes households from all 50 states and the District of Columbia and randomly selects adults (≥18 years) from sampled households for a detailed interview (N = 31 997). We identified 2622 adults aged 65 years or older who were enrolled in MA or TM plans, resulting in a weighted cohort of 14 222 243 adults, of whom 5 641 049 (39.7%) were enrolled in MA and 8 581 194 (60.3%) in TM. Race and ethnicity were determined according to self-report. For our main analysis, we defined adults with low income as those with a family income of less than or equal to 200% of the federal poverty level, representing families in the lowest one-sixth income level in the United States. In sensitivity analyses, we used alternate income thresholds to identify adults with low income. We intentionally used only the 2019 NHIS survey, which was redesigned compared with prior survey years, because it is the first survey year with low missingness rates for income.

### Outcomes

We evaluated self-reported outcomes across 4 domains: health care access, preventive care use, health care affordability, and prescription medication affordability. Health care access outcomes included having a usual place for medical care; a recent physician visit; cost-associated delays in medical care, dental care, and eye examinations; and not seeking medical care due to cost. Preventive care use outcomes included having recent diabetes screening, blood pressure screening, and cholesterol screening, as well as receiving an influenza vaccination in the past year. Health care affordability outcomes included being concerned about paying medical bills, problems paying them, or an inability to pay them (among individuals with problems paying medical bills). Prescription medication affordability outcomes included delaying or not filling prescriptions because of cost and skipping doses or taking less medication to save money.

### Statistical Analysis

We compared baseline characteristics for adults enrolled in MA vs TM, using the *t* test for continuous outcomes and the χ^2^ test for categorical outcomes. We fit survey-weighted logistic regression models to compare unadjusted outcomes among adults enrolled in MA vs TM. We then adjusted for the following variables in our models: patient demographic characteristics (age, sex, education, and race and ethnicity), self-reported clinical comorbid conditions (hypertension, hyperlipidemia, diabetes, history of myocardial infarction, history of stroke, history of cancer, chronic obstructive pulmonary disorder, dementia, asthma, and arthritis), and geography (region and rurality). We chose to adjust for clinical comorbid conditions because patients with MA and patients with TM may have a different burden of chronic conditions. We also adjusted for geographic factors because enrollment in plans may vary by US region. As a sensitivity analysis, we repeated this approach with alternate definitions of low income, including adults with family income less than or equal to 138% of the federal poverty level or less than or equal to 300% of the federal poverty level. Survey weights provided by the NHIS were applied to generate nationally representative estimates for all analyses.

Verbal informed consent was obtained by the NHIS interviewers. Adults with missing information for Medicare enrollment (0.3%), Medicare plan type (4.3%), and family income (0.0%) were excluded (<5%). The design of the NHIS has been reviewed and approved by the Centers for Disease Control and Prevention institutional review board. This study was based on secondary analyses of publicly available and deidentified NHIS data; therefore, no further institutional review board approval was sought from Beth Israel Deaconess Medical Center, in accordance with institutional policy. All analyses were conducted with R, version 4.0.3 (R Core Team), and survey weights were accounted for with the survey package. Two-sided *P* < .05 was used for statistical significance. No adjustment was performed for multiple testing. Data were analyzed from December 5, 2021, to April 10, 2022. Analytic scripts are available on request. This study followed Strengthening the Reporting of Observational Studies in Epidemiology (STROBE) reporting guidelines (eTable 7 in the Supplement).

## Results

The weighted study participants included 14 222 243 adults with low income who were aged 65 years or older (unweighted sample of 2622 adults), of whom 5 641 049 (39.7%) were enrolled in MA and 8 581 194 (60.3%) were enrolled in TM. The overall age of the cohort was 74.6 years (95% CI, 74.3-74.9). Between the MA and TM groups, the mean age (74.5 years [95% CI, 74.1-75.0] vs 74.7 years [95% CI, 74.3-75.1]; *P* = .63) and sex distribution (63.6% women [95% CI, 59.8%-67.3%] vs 60.4% women [95% CI, 57.4%-63.3%], *P* = .17; 36.4% men [95% CI, 32.7%-40.2%] vs 39.6% men [95% CI, 36.7%-42.6%], *P* = .17) were similar (Table 1). Compared with adults with low income in TM, adults with low income in MA were more likely to be non-Hispanic Asian (7.6% [95% CI, 5.0%-10.1%] vs 3.8% [95% CI, 2.4%-5.3%]; *P* = .01) or Hispanic (18.1% [95% CI, 14.3%-21.9%] vs 9.4% [95% CI, 7.2%-11.7%]; *P* < .001) and less likely to be White (56.0% vs 70.1%; *P* < .001). Adults with low income in MA were also less likely to be from rural areas (14.5% vs 30.6%; *P* < .001). The overall clinical comorbidity burden was generally similar between the 2 groups, although there were notable differences in plan enrollment by region (Northeast, 16.8% vs 17.1%; Midwest, 16.5% vs 21.9%; South, 42.6% vs 44.1%; West, 24.1% vs 16.8%; all *P* = .008) (Table 1). The income distribution of adults enrolled in MA vs TM is displayed in the Figure.

**Table 1.** Baseline Characteristics for Medicare Beneficiaries Aged 65 Years or Older With Low Income^a^^,^^b^

Characteristic	Beneficiaries, % (95% CI)		*P* value
	Medicare Advantage (N = 5 641 049)	Traditional Medicare (N = 8 581 194)	
Age, mean (95% CI), y	74.5 (74.1-75.0)	74.7 (74.3-75.1)	.63
Sex			
Male	36.4 (32.7-40.2)	39.6 (36.7-42.6)	.17
Female	63.6 (59.8-67.3)	60.4 (57.4-63.3)	.17
Race and ethnicity			
Hispanic	18.1 (14.3-21.9)	9.4 (7.2-11.7)	<.001
Non-Hispanic Asian	7.6 (5.0-10.1)	3.8 (2.4-5.3)	.01
Non-Hispanic Black	15.9 (12.8-19.1)	13.7 (11.1-16.2)	.24
Non-Hispanic White	56.0 (51.6-60.5)	70.1 (66.5-73.6)	<.001
Other^c^	2.4 (1.3-3.4)	3.0 (1.6-4.4)	.42
Clinical comorbid conditions			
Hypertension	67.3 (63.4-71.2)	69.0 (66.1-71.9)	.58
Hyperlipidemia	55.5 (51.6-59.4)	50.5 (47.4-53.5)	.05
Diabetes	25.2 (21.7-28.6)	27.0 (24.3-29.7)	.44
History of myocardial infarction	11.5 (9.1-13.8)	13.3 (11.2-15.5)	.26
History of stroke	10.4 (8.2-12.7)	13.5 (11.3-15.8)	.06
History of COPD	14.9 (12.4-17.4)	14.6 (12.7-16.5)	.85
History of cancer	22.6 (19.5-25.6)	21.8 (19.4-24.1)	.69
Region			
Northeast	16.8 (13.6-20.1)	17.1 (14.2-20.1)	.008
Midwest	16.5 (13.1-19.8)	21.9 (19.0-24.8)	
South	42.6 (37.9-47.3)	44.1 (40.5-47.7)	
West	24.1 (19.8-28.4)	16.8 (14.1-19.6)	
Rural	14.5 (11.3-17.6)	30.6 (27.1-34.1)	<.001
Bachelor degree or higher	10.1 (8.1-12.1)	9.1 (7.5-10.7)	.46

Abbreviation: COPD, chronic obstructive pulmonary disease.

^a^ Weighted estimates are national projections of US individuals aged 65 years or older who have traditional Medicare or Medicare Advantage.

^b^ The unweighted sample of adults aged 65 years or older with low income was 2622.

^c^ More specific race and ethnicity data were not available in the National Health Interview Survey. Race and ethnicity were determined by self-report.

**Figure.**
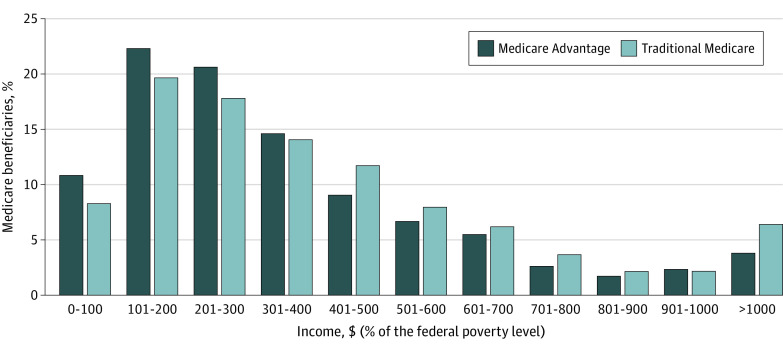
Income Distribution of Adults by Medicare Program Type Estimates are national projections of US individuals aged 65 years or older with traditional Medicare or Medicare Advantage.

### Health Care Access

Adults with low income in MA were more likely to have a usual place of care than those in TM (97.7% in MA vs 94.9% in TM; adjusted odds ratio [aOR], 2.37 [95% CI, 1.38-4.07]) but were similarly likely to have a recent physician visit (95.5% in MA vs 93.5% in TM; aOR, 1.39 [95% CI, 0.88-2.17]) and to delay medical care (5.3% in MA vs 5.7% in TM; aOR, 0.83 [95% CI, 0.56-1.24]) or not seek medical care (5.6% in MA vs 5.9% in TM; aOR, 0.86 [95% CI, 0.56-1.30]) due to costs. In addition, there were no significant differences in delaying dental care between MA and TM (27.9% in MA vs 28.2% in TM; aOR, 0.85 [95% CI, 0.66-1.09]) or eye examinations (66.6% in MA vs 64.7% in TM; aOR, 1.03 [95% CI, 0.82-1.30]) due to costs between these groups (Table 2).

**Table 2.** Health Care Access Among Medicare Beneficiaries With Low Income Covered by Medicare Advantage vs Traditional Medicare

Outcome	Beneficiaries, % (95% CI)		Odds ratio (95% CI)^a^		*P* value
	Medicare Advantage	Traditional Medicare	Unadjusted	Adjusted^b^	
Has a usual place for medical care	97.7 (96.7-98.6)	94.9 (93.6-96.1)	2.28 (1.38-3.75)	2.37 (1.38-4.07)	.002
Recent physician visit	95.5 (93.8-97.2)	93.5 (92.0-95.0)	1.47 (0.93-2.33)	1.39 (0.88-2.17)	.16
Delays in medical care due to cost	5.3 (3.9-6.8)	5.7 (4.3-7.1)	0.92 (0.63-1.35)	0.83 (0.56-1.24)	.37
Not seeking medical care due to cost	5.6 (4.1-7.2)	5.9 (4.5-7.3)	0.95 (0.64-1.40)	0.86 (0.56-1.30)	.47
Delays in dental care due to cost	27.9 (24.6-31.3)	28.2 (25.0-31.5)	0.99 (0.78-1.25)	0.85 (0.66-1.09)	.20
Delays in eye examinations due to cost	66.6 (62.7-70.5)	64.7 (61.7-67.6)	1.09 (0.88-1.35)	1.03 (0.82-1.30)	.80

^a^ Presented with traditional Medicare as the reference group.

^b^ Adjusted for patient demographic characteristics (age, sex, education, and race and ethnicity), self-reported clinical comorbid conditions (hypertension, hyperlipidemia, diabetes, history of myocardial infarction, history of stroke, history of cancer, chronic obstructive pulmonary disorder, dementia, asthma, and arthritis), and geography (region and rurality).

### Preventive Care Use

For measures of preventive care, adults with low income in MA were slightly more likely than those in TM to have undergone recent cholesterol screening (98.7% in MA vs 96.6% in TM; aOR, 2.58 [95% CI, 1.27-5.22]). However, there were no significant differences between these groups in the likelihood of recent diabetes screening (90.6% in MA vs 87.6% in TM; aOR, 1.21 [95% CI, 0.87-1.66]), blood pressure screening (96.8% in MA vs 95.2% in TM; aOR, 1.37 [95% CI, 0.84-2.23]), and receipt of an influenza vaccination in the past year (66.3% in MA vs 63.8% in TM; aOR, 1.16 [95% CI, 0.93-1.45]) (Table 3).

**Table 3.** Preventive Care Use Among Medicare Beneficiaries With Low Income Covered by Medicare Advantage vs Traditional Medicare

Outcome	Beneficiaries, % (95% CI)		Odds ratio (95% CI)^a^		*P* value
	Medicare Advantage	Traditional Medicare	Unadjusted	Adjusted^b^	
Recent diabetes screening	90.6 (88.5-92.7)	87.6 (85.7-89.6)	1.35 (1.01-1.82)	1.21 (0.87-1.66)	.26
Recent blood pressure screening	96.8 (95.6-97.9)	95.2 (94.1-96.4)	1.50 (0.96-2.33)	1.37 (0.84-2.23)	.21
Recent cholesterol screening	98.7 (98.0-99.4)	96.6 (95.5-97.7)	2.76 (1.48-5.16)	2.58 (1.27-5.22)	.009
Received influenza vaccination in past year	66.3 (62.5-70.2)	63.8 (60.8-66.8)	1.12 (0.90-1.38)	1.16 (0.93-1.45)	.18

^a^ Presented with traditional Medicare as the reference group.

^b^ Adjusted for patient demographic characteristics (age, sex, education, and race and ethnicity), self-reported clinical comorbid conditions (hypertension, hyperlipidemia, diabetes, history of myocardial infarction, history of stroke, history of cancer, chronic obstructive pulmonary disorder, dementia, asthma, and arthritis), and geography (region and rurality).

### Health Care and Prescription Medication Affordability

We observed no significant differences between adults with low income who were enrolled in MA vs those who were enrolled in TM regarding the likelihood of being concerned about paying medical bills (47.3% in MA vs 44.2% in TM; aOR, 1.09 [95% CI, 0.88-1.35]) or having problems paying medical bills (17.1% in MA vs 17.2% in TM; aOR, 0.94 [95% CI, 0.69-1.27]). Among those having problems paying medical bills, adults with low income who were enrolled in MA and those enrolled in TM were similarly likely to report inability to pay medical bills (57.8% in MA vs 60.5% in TM; aOR, 0.76 [95% CI, 0.45-1.29]).

Adults with low income who were enrolled in MA and those enrolled in TM were also similarly likely to delay filling prescriptions (7.4% in MA vs 6.2% in TM; aOR, 1.22 [95% CI, 0.78-1.92]) or not fill prescriptions (7.8% in MA vs 7.4% in TM; aOR, 1.01 [95% CI, 0.70-1.45]) due to costs. In addition, there were no significant differences between these 2 groups in the likelihood of skipping doses (5.4% in MA vs 4.5% in TM; aOR, 1.16 [95% CI, 0.70-1.92]) or taking less medication (6.6% in MA vs 5.4% in TM; aOR, 1.16 [95% CI, 0.73-1.85]) due to costs (Table 4).

**Table 4.** Health Care and Prescription Medication Affordability for Medicare Beneficiaries With Low Income Covered by Medicare Advantage vs Traditional Medicare

Outcome	Beneficiaries, % (95% CI)		Odds ratio (95% CI)^a^		*P* value
	Medicare Advantage	Traditional Medicare	Unadjusted	Adjusted^b^	
Health care affordability					
Concerned about paying medical bills	47.3 (43.4-51.1)	44.2 (41.0-47.5)	1.13 (0.92-1.38)	1.09 (0.88-1.35)	.45
Problems paying medical bills	17.1 (14.3-19.9)	17.2 (14.6-19.7)	1.00 (0.77-1.30)	0.94 (0.69-1.27)	.69
Inability to pay medical bills	57.8 (48.6-66.9)	60.5 (52.3-68.7)	0.89 (0.54-1.48)	0.76 (0.45-1.29)	.32
Prescription medication affordability					
Delays in filling prescriptions due to cost	7.4 (5.2-9.7)	6.2 (4.7-7.6)	1.23 (0.81-1.86)	1.22 (0.78-1.92)	.38
Not filling prescriptions due to cost	7.8 (5.7-9.9)	7.4 (6.0-8.8)	1.06 (0.75-1.50)	1.01 (0.70-1.45)	.96
Skipping doses of needed medications to save money	5.4 (3.6-7.2)	4.5 (3.3-5.8)	1.20 (0.76-1.90)	1.16 (0.70-1.92)	.57
Taking less medication to save money	6.6 (4.5-8.7)	5.4 (4.0-6.8)	1.25 (0.81-1.92)	1.16 (0.73-1.85)	.54

^a^ Presented with traditional Medicare as the reference group.

^b^ Adjusted for patient demographic characteristics (age, sex, education, and race and ethnicity), self-reported clinical comorbid conditions (hypertension, hyperlipidemia, diabetes, history of myocardial infarction, history of stroke, history of cancer, chronic obstructive pulmonary disorder, dementia, asthma, and arthritis), and geography (region and rurality).

### Sensitivity Analyses

We performed sensitivity analyses using 2 alternate specifications to identify adults with low income and observed largely similar findings, which are shown in eTables 1 through 6 in the Supplement. However, when the cohort with low income was more inclusive (using 300% of the federal poverty level as the income cutoff as opposed to 200% in the primary analysis), we found that adults enrolled in MA were also more likely to have a recent physician visit, diabetes screening, and blood pressure screening than those enrolled in TM (eTable 2 in the Supplement), although absolute percentage-point differences between the cohorts were small (<3% difference for each outcome).

## Discussion

We found little evidence that key measures of health care access and affordability differ between adults with low income who are enrolled in MA and those enrolled in TM. Although MA enrollment was associated with a slightly higher likelihood of having a usual place of care and recent cholesterol screening than TM enrollment, the proportion of adults with low income who delayed or avoided medical, dental, or vision care due to cost was similar between both plans. In addition, there were no significant differences in cost-associated barriers to prescription drugs between adults with low income who were enrolled in MA and those who were enrolled in TM.

Medicare Advantage is widely thought to cost the federal government more than TM per beneficiary,^15^ and 1 potential interpretation of our findings is that MA does not provide benefits commensurate with the increased costs, specifically among adults with low income. Although MA plans systematically track performance on quality measures, which are tied to star ratings and reimbursement,^16^ we found that these plans performed better than TM on only 2 of 17 studied measures (usual place of care and recent cholesterol screening), and absolute differences between these groups were small. Furthermore, although a primary feature of MA plans is the supplemental benefits, particularly vision and dental coverage,^17^ we did not observe any differences in the proportion of adults who delayed dental care or eye examinations because of cost. Indeed, many beneficiaries in both TM and MA continue to experience difficulty accessing dental and vision care (ie, 2 of 3 adults in either plan delayed eye examinations due to cost), significantly more than the number of beneficiaries who experience difficulty accessing other forms of medical care, such as prescription drugs. One possibility is that MA participants may be less aware of these supplemental benefits.

Another possible interpretation is that MA plans can achieve similar or slightly better metrics of access, preventive care, and affordability for beneficiaries with low income compared with TM plans, while enrolling a higher proportion of beneficiaries from racial and ethnic minority groups.^18^ In our study, MA beneficiaries with low income were approximately twice as likely as TM beneficiaries to be Hispanic adults, a growing demographic segment that faces important barriers to health care and has historically experienced poor access.^19^ The higher rates of MA enrollment among adults in racial and ethnic minority groups may reflect greater market penetration of MA plans in diverse communities. Our study builds on recent work in the broader set of Medicare beneficiaries, which found that beneficiaries in racial and ethnic minority groups with MA may experience better access to care than those enrolled in TM.^20^

Another potential cause for the lack of differences in most outcome measures may be that adults with low income in both MA and TM groups face substantial upstream barriers that make health care inaccessible or unaffordable regardless of their health insurance benefits. For example, adults with low income tend to have poor access to transportation, have less stable housing, face increased social discrimination, and have lower health literacy, all factors that may affect their ability to access health care.^21^ Although improving health insurance for adults with low income is 1 important avenue to improve health disparities, upstream efforts to address the structural factors underpinning such disparities are also required.

Although our study is among the first, to our knowledge, to focus on beneficiaries with low income, our findings have some similarities with prior studies of the entire cohort of Medicare beneficiaries. In 1 analysis of 2018 Medicare data, a similar proportion of beneficiaries with TM (5%) and MA (7%) reported problems obtaining needed health care, with approximately one-third in both groups reporting high costs as a barrier.^22^ This study also found that MA beneficiaries were slightly more likely to have a usual source of care, similar to our analysis, and receive more robust care management services. Given that MA plans receive risk-adjusted per-member payments from the federal government and retain a portion of dollars not spent on medical claims as profits,^23^ an increased emphasis on preventive care might be expected in MA. We observed largely similar use of preventive care services between adults with low income in MA and those in TM, suggesting this incentive may generate only a modest benefit specifically among adults with low income. However, in our sensitivity analysis that used a less restrictive income cutoff (<300% of the federal poverty level), we observed higher use of diabetes, cholesterol, and blood pressure screening in MA compared with TM, although absolute differences between these plans were modest (<3% difference for each outcome).

The insurance coverage and benefits offered by most MA plans are often more comprehensive than those offered by TM alone, but more than 80% of Medicare beneficiaries have some form of supplemental insurance.^24^ For individuals with low income, this supplemental insurance often takes the form of Medicaid for those dually eligible, whereas others elect to purchase supplemental coverage (Medigap) to augment the TM benefit structure (eg, the Part D coverage gap, or “donut hole”). Other individuals may have access to employer-sponsored insurance as supplemental coverage.^25^ The prevalence of supplemental coverage in TM may explain why we found comparable levels of health care access and affordability between MA and TM for individuals with low income. Prior work that has focused on all beneficiaries—not just those with low income—has found that cost-associated problems were slightly less prevalent among beneficiaries in TM vs MA and were associated with supplemental coverage for TM beneficiaries.^26^

### Limitations

This study has limitations. First, although we compared adults with low income in MA vs TM after accounting for demographic characteristics, clinical comorbid conditions, and geography, our analysis may not have captured other variables associated with Medicare program type and access or affordability. A strength of our approach, however, is that we focused on a subset of beneficiaries who were likely to be more similar across plans (adults with low income), particularly regarding socioeconomic factors. Second, there is considerable variability in plan design within MA.^27^ Differences in enrollment by state or in the availability or affordability of additional coverage by location, besides regional differences, could not be assessed. Our analysis focused on providing a nationwide assessment of MA vs TM, but it is possible that some individual MA plans perform better or worse than others on measures of access and affordability, and further study is needed to evaluate differences between MA plans. Third, although the proportions of adults with MA vs TM who were dually enrolled in Medicare and Medicaid were identical in our study cohort, we did not include this variable in our models given that reports of dual enrollment may be undercaptured in the NHIS.^28^ Fourth, prior studies have shown that separating special needs plans from other MA plans can help improve comparisons of TM vs MA, and we were unable to do so with the available data.^22^ Fifth, our study used survey data, which may be subject to response bias.^29^ However, we believe a strength of such self-reported survey data is that it mitigates concerns regarding well-documented differences in coding intensity between MA and TM plans when claims-based data are used.

## Conclusions

In this study of Medicare beneficiaries in 2019, we found largely no differences across key measures of health care access and preventive care for adults with low income who were enrolled in MA compared with those who were enrolled in TM. In addition, cost-associated barriers to medical care and prescription drugs were similar between these groups. Despite more generous benefits and stronger incentives to coordinate care and manage health, MA plans may not provide meaningful improvements in access, affordability, or preventive care compared with TM for adults with low income. These findings suggest that the increasing enrollment of adults with low income in MA may not meaningfully advance health equity in the Medicare program.

## Notes

Supplement. **eTable 1.** Health Care Access Among Medicare Beneficiaries With Low Income Covered by Medicare Advantage vs Traditional Medicare Using an Alternative Definition for Low Income at 300% or Less of the Federal Poverty Level **eTable 2.** Preventive Care Use Among Medicare Beneficiaries With Low Income Covered by Medicare Advantage vs Traditional Medicare Using an Alternative Definition for Low Income at 300% or Less of the Federal Poverty Level **eTable 3.** Health Care and Prescription Medication Affordability Among Medicare Beneficiaries With Low Income Covered by Medicare Advantage vs Traditional Medicare Using an Alternative Definition for Low Income at 300% or Less of the Federal Poverty Level **eTable 4.** Health Care Access Among Medicare Beneficiaries With Low Income Covered by Medicare Advantage vs Traditional Medicare Using an Alternative Definition for Low Income at 138% or Less of the Federal Poverty Level **eTable 5.** Preventive Care Use Among Medicare Beneficiaries With Low Income Covered by Medicare Advantage vs Traditional Medicare Using an Alternative Definition for Low Income at 138% or Less of the Federal Poverty Level **eTable 6.** Health Care and Prescription Medication Affordability Among Medicare Beneficiaries With Low Income Covered by Medicare Advantage vs Traditional Medicare Using an Alternative Definition for Low Income at 138% or Less of the Federal Poverty Level **eTable 7.** STROBE Statement
