# Supplementary material for: Comparison of Medicare Advantage vs Traditional Medicare for Health Care Access, Affordability, and Use of Preventive Services Among Adults With Low Income
Source: JAMA Netw Open. 2022 Jun 7;5(6):e2215227. doi: 10.1001/jamanetworkopen.2022.15227 (PMC9175080; doi:10.1001/jamanetworkopen.2022.15227)
Supplement: Supplement. — eTable 1. Health Care Access Among Medicare Beneficiaries With Low Income Covered by Medicare Advantage vs Traditional Medicare Using an Alternative Definition for Low Income at 300% or Less of the Federal Poverty Level eTable 2. Preventive Care Use Among Medicare Beneficiaries With Low Income Covered by Medicare Advantage vs Traditional Medicare Using an Alternative Definition for Low Income at 300% or Less of the Federal Poverty Level eTable 3. Health Care and Prescription Medication Affordability Among Medicare Beneficiaries With Low Income Covered by Medicare Advantage vs Traditional Medicare Using an Alternative Definition for Low Income at 300% or Less of the Federal Poverty Level eTable 4. Health Care Access Among Medicare Beneficiaries With Low Income Covered by Medicare Advantage vs Traditional Medicare Using an Alternative Definition for Low Income at 138% or Less of the Federal Poverty Level eTable 5. Preventive Care Use Among Medicare Beneficiaries With Low Income Covered by Medicare Advantage vs Traditional Medicare Using an Alternative Definition for Low Income at 138% or Less of the Federal Poverty Level eTable 6. Health Care and Prescription Medication Affordability Among Medicare Beneficiaries With Low Income Covered by Medicare Advantage vs Traditional Medicare Using an Alternative Definition for Low Income at 138% or Less of the Federal Poverty Level eTable 7. STROBE Statement [file jamanetwopen-e2215227-s001.pdf]

Supplemental Online Content

Aggarwal R, Gondi S, Wadhera RK. Comparison of Medicare Advantage vs traditional Medicare for health care access, affordability, and use of preventive services among adults with low income. *JAMA Netw Open*. 2022;5(6):e2215227. doi:10.1001/jamanetworkopen.2022.15227

eTable 1. Health Care Access Among Medicare Beneficiaries With Low Income Covered by Medicare Advantage vs Traditional Medicare Using an Alternative Definition for Low Income at 300% or Less of the Federal Poverty Level

eTable 2. Preventive Care Use Among Medicare Beneficiaries With Low Income Covered by Medicare Advantage vs Traditional Medicare Using an Alternative Definition for Low Income at 300% or Less of the Federal Poverty Level

eTable 3. Health Care and Prescription Medication Affordability Among Medicare Beneficiaries With Low Income Covered by Medicare Advantage vs Traditional Medicare Using an Alternative Definition for Low Income at 300% or Less of the Federal Poverty Level

eTable 4. Health Care Access Among Medicare Beneficiaries With Low Income Covered by Medicare Advantage vs Traditional Medicare Using an Alternative Definition for Low Income at 138% or Less of the Federal Poverty Level

eTable 5. Preventive Care Use Among Medicare Beneficiaries With Low Income Covered by Medicare Advantage vs Traditional Medicare Using an Alternative Definition for Low Income at 138% or Less of the Federal Poverty Level

eTable 6. Health Care and Prescription Medication Affordability Among Medicare Beneficiaries With Low Income Covered by Medicare Advantage vs Traditional Medicare Using an Alternative Definition for Low Income at 138% or Less of the Federal Poverty Level

eTable 7. STROBE Statement

This supplemental material has been provided by the authors to give readers additional information about their work.

© 2022 Aggarwal R et al. *JAMA Network Open.*

eTable 1. Health Care Access Among Medicare Beneficiaries With Low Income Covered by Medicare Advantage vs Traditional Medicare Using an Alternative Definition for Low Income at 300% or Less of the Federal Poverty Level

	**Medicare Advantage**	**Traditional Medicare**	**Unadjusted Odds Ratio**[1] (95% Confidence Interval)	**Adjusted Odds Ratio**[1] (95% Confidence Interval)	**P-Value**
**Has a Usual Place for Medical Care**	97.9% (97.2%, 98.6%)	95.5% (94.5%, 96.5%)	2.18 (1.44, 3.30)	2.45 (1.58, 3.81)	<0.001
**Recent Physician Visit**	96.3% (95.1%, 97.4%)	94.7% (93.6%, 95.7%)	1.46 (1.00, 2.14)	1.52 (1.04, 2.22)	0.03
**Delays in Medical Care Due to Cost**	4.8% (3.6%, 5.9%)	4.6% (3.5%, 5.6%)	1.05 (0.75, 1.47)	1.02 (0.73, 1.43)	0.91
**Not Seek Medical Care Due to Cost**	4.8% (3.6%, 6.0%)	4.6% (3.6%, 5.6%)	1.05 (0.75, 1.48)	0.98 (0.68, 1.40)	0.90
**Delays in Dental Care Due to Cost**	24.8% (22.3%, 27.4%)	24.4% (21.9%, 26.8%)	1.02 (0.85, 1.23)	0.90 (0.74, 1.11)	0.33
**Delays in Eye Examinations Due to Cost**	69.0% (66.1%, 71.8%)	67.2% (64.9%, 69.4%)	1.09 (0.92, 1.29)	1.07 (0.90, 1.28)	0.43

[1] Odds ratios compare low-income adults enrolled in Medicare Advantage to those enrolled in Traditional Medicare (reference group)

This supplemental material has been provided by the authors to give readers additional information about their work.

© 2022 Aggarwal R et al. *JAMA Network Open.*

eTable 2. Preventive Care Use Among Medicare Beneficiaries With Low Income Covered by Medicare Advantage vs Traditional Medicare Using an Alternative Definition for Low Income at 300% or Less of the Federal Poverty Level

	**Medicare Advantage**	**Traditional Medicare**	**Unadjusted Odds Ratio[1]** (95% Confidence Interval)	**Adjusted Odds Ratio[1]** (95% Confidence Interval)	**P-Value**
**Recent Diabetes Screening**	91.6% (89.9%, 93.2%)	88.6% (87.1%, 90.1%)	1.39 (1.07, 1.81)	1.37 (1.05, 1.79)	0.02
**Recent Blood Pressure Screening**	97.4% (96.6%, 98.2%)	95.9% (95.1%, 96.7%)	1.60 (1.11, 2.31)	1.70 (1.14, 2.54)	0.009
**Recent Cholesterol Screening**	98.8% (98.3%, 99.3%)	97.0% (96.2%, 97.8%)	2.63 (1.58, 4.37)	2.74 (1.56, 4.80)	<0.001
**Received Influenza Vaccination in Last One Year**	69.5% (66.5%, 72.4%)	66.2% (63.9%, 68.5%)	1.16 (0.98, 1.38)	1.18 (0.99, 1.41)	0.06

[1] Odds ratios compare low-income adults enrolled in Medicare Advantage to those enrolled in Traditional Medicare (reference group)

This supplemental material has been provided by the authors to give readers additional information about their work.

© 2022 Aggarwal R et al. *JAMA Network Open.*

eTable 3. Health Care and Prescription Medication Affordability Among Medicare Beneficiaries With Low Income Covered by Medicare Advantage vs Traditional Medicare Using an Alternative Definition for Low Income at 300% or Less of the Federal Poverty Level

	**Medicare Advantage**	**Traditional Medicare**	**Unadjusted Odds Ratio**[1] (95% Confidence Interval)	**Adjusted Odds Ratio**[1] (95% Confidence Interval)	**P-Value**
**Health Care Affordability**					
**Worry about Paying Medical Bills**	45.2% (42.3%, 48.1%)	41.3% (38.9%, 43.8%)	1.17 (1.00, 1.37)	1.14 (0.96, 1.34)	0.13
**Problems Paying Medical Bills**	14.6% (12.5%, 16.6%)	14.7% (12.8%, 16.6%)	0.99 (0.80, 1.23)	0.96 (0.75, 1.22)	0.73
**Inability to Pay Medical Bills**[2]	55.0% (46.8%, 63.2%)	58.3% (51.3%, 65.2%)	0.87 (0.56, 1.36)	0.74 (0.45, 1.20)	0.22
**Prescription Medication Affordability**					
**Delays in Filling Prescriptions Due to Cost**	7.1% (5.4%, 8.8%)	5.3% (4.2%, 6.4%)	1.38 (0.98, 1.94)	1.38 (0.95, 1.99)	0.09
**Not Filling Prescriptions Due to Cost**	7.0% (5.4%, 8.6%)	6.3% (5.1%, 7.4%)	1.14 (0.85, 1.53)	1.11 (0.82, 1.50)	0.52
**Skipping Doses of Needed Medications to Save Money**	5.0% (3.7%, 6.3%)	3.8% (2.9%, 4.7%)	1.33 (0.91, 1.93)	1.25 (0.83, 1.89)	0.28
**Taking Less Medication to Save Money**	5.7% (4.1%, 7.2%)	4.9% (3.7%, 6.1%)	1.16 (0.80, 1.70)	1.04 (0.70, 1.54)	0.84

[1] Odds ratios compare low-income adults enrolled in Medicare Advantage to those enrolled in Traditional Medicare (reference group)

[2] Among individuals with problems paying medical bills

This supplemental material has been provided by the authors to give readers additional information about their work.

© 2022 Aggarwal R et al. *JAMA Network Open.*

eTable 4. Health Care Access Among Medicare Beneficiaries With Low Income Covered by Medicare Advantage vs Traditional Medicare Using an Alternative Definition for Low Income at 138% or Less of the Federal Poverty Level

	**Medicare Advantage**	**Traditional Medicare**	**Unadjusted Odds Ratio**[1] (95% Confidence Interval)	**Adjusted Odds Ratio**[1] (95% Confidence Interval)	**P-Value**
**<u>Health Care Access</u>**					
**Has a Usual Place for Medical Care**	97.2% (95.8%, 98.5%)	94.1% (92.2%, 96%)	2.17 (1.18, 3.99)	2.12 (1.06, 4.22)	0.03
**Recent Physician Visit**	95.3% (92.7%, 97.9%)	93.3% (91.5%, 95.2%)	1.44 (0.74, 2.81)	1.20 (0.65, 2.21)	0.56
**Delays in Medical Care Due to Cost**	6.4% (4.1%, 8.6%)	7.2% (5.0%, 9.3%)	0.88 (0.54, 1.44)	0.80 (0.47, 1.36)	0.40
**Not Seek Medical Care Due to Cost**	6.1% (3.8%, 8.4%)	8.1% (5.7%, 10.4%)	0.74 (0.44, 1.24)	0.68 (0.39, 1.19)	0.18
**Delays in Dental Care Due to Cost**	31.9% (26.8%, 37.1%)	28.8% (24.8%, 32.8%)	1.16 (0.85, 1.58)	1.01 (0.73, 1.40)	0.95
**Delays in Eye Examinations Due to Cost**	65.1% (59.8%, 70.4%)	63.5% (59.7%, 67.3%)	1.07 (0.81, 1.42)	0.99 (0.73, 1.33)	0.93

[1] Odds ratios compare low-income adults enrolled in Medicare Advantage to those enrolled in Traditional Medicare (reference group)

This supplemental material has been provided by the authors to give readers additional information about their work.

© 2022 Aggarwal R et al. *JAMA Network Open.*

eTable 5. Preventive Care Use Among Medicare Beneficiaries With Low Income Covered by Medicare Advantage vs Traditional Medicare Using an Alternative Definition for Low Income at 138% or Less of the Federal Poverty Level

	**Medicare Advantage**	**Traditional Medicare**	**Unadjusted Odds Ratio**[1] (95% Confidence Interval)	**Adjusted Odds Ratio**[1] (95% Confidence Interval)	**P-Value**
**Primary and Preventive Care**					
**Recent Diabetes Screening**	90.5% (87.7%, 93.2%)	88.7% (86.4%, 91.0%)	1.20 (0.81, 1.78)	0.91 (0.60, 1.39)	0.66
**Recent Blood Pressure Screening**	96.6% (95.1%, 98.2%)	94.8% (93.2%, 96.5%)	1.56 (0.87, 2.81)	1.17 (0.60, 2.29)	0.64
**Recent Cholesterol Screening**	98.9% (98.1%, 99.7%)	95.8% (94.0%, 97.5%)	3.97 (1.64, 9.63)	3.03 (1.06, 8.70)	0.04
**Received Influenza Vaccination in Last One Year**	64.7% (59.2%, 70.2%)	61.1% (57.4%, 64.7%)	1.17 (0.88, 1.55)	1.25 (0.94, 1.67)	0.12

[1] Odds ratios compare low-income adults enrolled in Medicare Advantage to those enrolled in Traditional Medicare (reference group)

This supplemental material has been provided by the authors to give readers additional information about their work.

© 2022 Aggarwal R et al. *JAMA Network Open.*

eTable 6. Health Care and Prescription Medication Affordability Among Medicare Beneficiaries With Low Income Covered by Medicare Advantage vs Traditional Medicare Using an Alternative Definition for Low Income at 138% or Less of the Federal Poverty Level

	**Traditional Medicare**	**Medicare Advantage**	**Unadjusted Odds Ratio[1]** (95% Confidence Interval)	**Adjusted Odds Ratio[1]** (95% Confidence Interval)	**P-Value**
**Health Care Affordability**					
**Worry about Paying Medical Bills**	45.9% (40.5%, 51.3%)	46.0% (41.6%, 50.4%)	0.99 (0.76, 1.31)	0.88 (0.66, 1.18)	0.39
**Problems Paying Medical Bills**	18.2% (13.9%, 22.5%)	19% (15.7%, 22.4%)	0.95 (0.67, 1.34)	0.85 (0.56, 1.30)	0.47
**Inability to Pay Medical Bills[2]**	65.0% (53%, 77%)	62.9% (52.6%, 73.1%)	1.09 (0.55, 2.18)	0.97 (0.47, 1.99)	0.93
**Prescription Medication Affordability**					
**Delays in Filling Prescriptions Due to Cost**	9.2% (5.9%, 12.6%)	7.3% (5.0%, 9.6%)	1.29 (0.75, 2.21)	1.28 (0.73, 2.26)	0.39
**Not Filling Prescriptions Due to Cost**	9.5% (6.3%, 12.8%)	7.7% (5.5%, 10.0%)	1.25 (0.78, 2.02)	1.13 (0.69, 1.85)	0.62
**Skipping Doses of Needed Medications to Save Money**	6.8% (4%, 9.5%)	4.8% (2.9%, 6.7%)	1.45 (0.78, 2.69)	1.42 (0.70, 2.87)	0.33
**Taking Less Medication to Save Money**	8.0% (4.8%, 11.1%)	5.8% (3.6%, 7.9%)	1.41 (0.78, 2.57)	1.31 (0.70, 2.46)	0.40

[1] Odds ratios compare low-income adults enrolled in Medicare Advantage to those enrolled in Traditional Medicare (reference group)

[2] Among individuals with problems paying medical bills

This supplemental material has been provided by the authors to give readers additional information about their work.

© 2022 Aggarwal R et al. *JAMA Network Open.*

eTable 7. STROBE Statement

	Item No	Recommendation	Page No
**Title and abstract**	1	(*a*) Indicate the study's design with a commonly used term in the title or the abstract	3
		(*b*) Provide in the abstract an informative and balanced summary of what was done and what was found	3-4
**Introduction**			
Background/rationale	2	Explain the scientific background and rationale for the investigation being reported	5-6
Objectives	3	State specific objectives, including any prespecified hypotheses	5
**Methods**			
Study design	4	Present key elements of study design early in the paper	6-8
Setting	5	Describe the setting, locations, and relevant dates, including periods of recruitment, exposure, follow-up, and data collection	6-7
Participants	6	(*a*) Give the eligibility criteria, and the sources and methods of selection of participants	6-7
Variables	7	Clearly define all outcomes, exposures, predictors, potential confounders, and effect modifiers. Give diagnostic criteria, if applicable	7-8
Data sources/ measurement	8*	For each variable of interest, give sources of data and details of methods of assessment (measurement). Describe comparability of assessment methods if there is more than one group	*6-7*
Bias	9	Describe any efforts to address potential sources of bias	8
Study size	10	Explain how the study size was arrived at	6-7,9
Quantitative variables	11	Explain how quantitative variables were handled in the analyses. If applicable, describe which groupings were chosen and why	8
Statistical methods	12	(*a*) Describe all statistical methods, including those used to control for confounding	8
		(*b*) Describe any methods used to examine subgroups and interactions	8
		(*c*) Explain how missing data were addressed	8
		(*d*) If applicable, describe analytical methods taking account of sampling strategy	8
		(*e*) Describe any sensitivity analyses	8

This supplemental material has been provided by the authors to give readers additional information about their work.

**Results**			
Participants	13*	(a) Report numbers of individuals at each stage of study—eg numbers potentially eligible, examined for eligibility, confirmed eligible, included in the study, completing follow-up, and analysed	6,9
		(b) Give reasons for non-participation at each stage	NA
		(c) Consider use of a flow diagram	NA
Descriptive data	14*	(a) Give characteristics of study participants (eg demographic, clinical, social) and information on exposures and potential confounders	9, 18-19
		(b) Indicate number of participants with missing data for each variable of interest	8
Outcome data	15*	Report numbers of outcome events or summary measures	9-10
Main results	16	(*a*) Give unadjusted estimates and, if applicable, confounder-adjusted estimates and their precision (eg, 95% confidence interval). Make clear which confounders were adjusted for and why they were included	20-22
		(*b*) Report category boundaries when continuous variables were categorized	NA
		(*c*) If relevant, consider translating estimates of relative risk into absolute risk for a meaningful time period	NA
Other analyses	17	Report other analyses done—eg analyses of subgroups and interactions, and sensitivity analyses	10, Appendix
**Discussion**			
Key results	18	Summarise key results with reference to study objectives	11
Limitations	19	Discuss limitations of the study, taking into account sources of potential bias or imprecision. Discuss both direction and magnitude of any potential bias	14-15
Interpretation	20	Give a cautious overall interpretation of results considering objectives, limitations, multiplicity of analyses, results from similar studies, and other relevant evidence	11
Generalisability	21	Discuss the generalisability (external validity) of the study results	11
**Other information**			
Funding	22	Give the source of funding and the role of the funders for the present study and, if applicable, for the original study on which the present article is based	17

*Give information separately for exposed and unexposed groups.

This supplemental material has been provided by the authors to give readers additional information about their work.

© 2022 Aggarwal R et al. *JAMA Network Open.*

Note: An Explanation and Elaboration article discusses each checklist item and gives methodological background and published examples of transparent reporting. The STROBE checklist is best used in conjunction with this article (freely available on the Web sites of PLoS Medicine at http://www.plosmedicine.org/, Annals of Internal Medicine at http://www.annals.org/, and Epidemiology at http://www.epidem.com/). Information on the STROBE Initiative is available at www.strobe-statement.org.

This supplemental material has been provided by the authors to give readers additional information about their work.

© 2022 Aggarwal R et al. *JAMA Network Open.*
